# Preoperative Resilience Does Not Predict Functional Outcomes After Rotator Cuff Repair: A Systematic Review

**DOI:** 10.1002/ars2.70025

**Published:** 2026-05-07

**Authors:** Paxton Sweeney, Nzubechukwu (Joshua) Obidike, Ryan W. Paul, Jordan T. Windsor, Francis J. Sirch, Fotios P. Tjoumakaris, Kevin B. Freedman

**Affiliations:** ^1^ Hackensack Meridian School of Medicine Nutley New Jersey U.S.A.; ^2^ Department of Orthopedic Surgery Monmouth Medical Center Long Branch New Jersey U.S.A.; ^3^ Rothman Orthopaedic Institute Philadelphia Pennsylvania U.S.A.

## Abstract

**Purpose:**

To determine whether preoperative or concurrent resilience is related to postoperative functional outcomes after arthroscopic rotator cuff repair (aRCR).

**Methods:**

A literature search was conducted from PubMed, SportDiscus, and OVID Medline databases to identify studies evaluating the effect of preoperative resilience on postoperative outcomes in aRCR patients. Articles were included if they measured preoperative or concurrent resilience in aRCR patients as well as postoperative outcomes with a minimum 6‐month follow‐up. Concurrent resilience refers to resilience measured during the postoperative recovery period rather than prior to surgery (preoperative resilience). Patient resilience as measured via any resilience scale (e.g., Brief Resilience Scale, Life Orientation Test‐Revised, and Connor‐Davidson Resilience Scale) were collected, as were functional outcome scores such as the American Shoulder and Elbow Surgeons (ASES) score. Due to heterogeneity in study designs, a qualitative analysis was performed.

**Results:**

Seven studies, with a total of 614 patients, met the inclusion criteria. Six included studies that evaluated preoperative patient resilience and ASES scores found that preoperative patient resilience was not correlated with ASES scores 0.5 to 4 years following aRCR. Four studies assessed the relationship between concurrent resilience and ASES scores, with 2 studies showing significant correlations and 2 studies finding no significant correlation.

**Conclusions:**

Preoperative patient resilience does not correlate with postoperative ASES scores after aRCR. Concurrent patient resilience during recovery shows mixed associations with postoperative ASES scores, with some studies reporting significant correlations and others finding none.

**Level of Evidence:**

Level IV, systematic review of Level II to IV studies.

## Graphical abstract

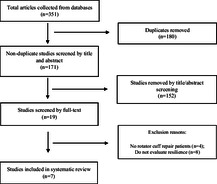

Resilience is a dynamic psychological construct reflecting an individual's capacity to adapt, withstand, and recover from stressors or adversity, including injury, pain, and the demands of surgery.^1^ Resilience helps patients manage the stress associated with surgical rehabilitation and has been investigated as a potential factor influencing recovery after orthopaedic surgery.^2^ Whereas some studies have shown that higher resilience is linked to better postoperative outcomes across various orthopaedic surgeries such as total knee arthroplasty, total shoulder arthroplasty, rotator cuff repair (RCR), and lumbar fusion surgery, other research has found weak, inconsistent, or no association between preoperative resilience and functional recovery.^3^, ^4^, ^5^, ^6^, ^7^, ^8^, ^9^ Patients with more resilience may experience less pain, achieve quicker functional recoveries, and report higher satisfaction levels after surgery, but these effects are not universally observed and may be influenced by confounding factors such as age, comorbidities, and baseline function.^3^, ^5^, ^6^, ^7^, ^8^, ^9^

Otlans et al.^1^ highlighted that resilience in orthopaedic patients may serve as a predictor of postoperative success and could be integrated into clinical assessments to identify patients who may benefit from targeted psychological interventions. Conversely, DeFoor et al.^9^ concluded that current evidence does not support resilience as a definitive determinant of surgical outcome, citing methodological heterogeneity and mixed findings across studies.

Within the context of arthroscopic RCR (aRCR), existing literature is similarly mixed—some studies have reported that higher resilience is correlated with better functional outcomes and lower postoperative pain levels, whereas others have found no significant relationship.^5^, ^6^, ^10^, ^11^, ^12^, ^13^, ^14^ Given this uncertainty, further investigation is warranted to clarify whether resilience meaningfully impacts recovery in this population.

The purpose of this study was to determine whether preoperative or concurrent resilience is related to postoperative functional outcomes after aRCR. The authors hypothesized that patients with higher preoperative resilience would experience better functional outcomes than patients with lower preoperative resilience.

## METHODS

This study was conducted according to Preferred Reporting Items for Systematic Reviews and Meta‐Analysis guidelines.^15^ A literature search was performed using the PubMed, SportDiscus, and Medline (OVID) databases from inception until June 2024 to identify original research studies that evaluate the effects of preoperative patient resilience on postoperative aRCR outcomes using the following search terms: ((rotator cuff repair) OR (RCR) OR (rotator cuff) OR (shoulder) OR (arthroscopy)) AND ((resilient) OR (resilience).

### Inclusion/Exclusion Criteria

Two coinvestigators (P.S., N.O.) screened the studies by title/abstract to confirm studies evaluated aRCR and resilience levels. The remaining studies underwent full‐text screening by the same 2 coinvestigators to confirm that preoperative resilience and postoperative aRCR outcomes were evaluated. Any discrepancies were resolved by a third coinvestigator (R.P.). Studies that were original research studies, evaluated patient resilience, evaluated postoperative clinical or patient‐reported outcomes, included 10 or more patients, and had a minimum 6‐month follow‐up time were included. Eligibility criteria included studies of adult patients undergoing aRCR for partial‐thickness, full‐thickness, or mixed tear types, as reported by the original study authors. Studies were eligible regardless of whether tear type was stratified in the results, but reporting of tear type was extracted when available. Studies that did not evaluate aRCR, preoperative patient resilience, and postoperative outcomes and did not have patient follow‐up of at least 6 months were excluded.

The following demographic variables were collected: author, year of publication, level of evidence, sample size, patient sex, patient age, tear size, smoking status, rehabilitation protocol, and repair method. Patient resilience as measured via any resilience scale (e.g., Brief Resilience Scale [BRS; Supporting Information],^16^ Connor‐Davidson Resilience Scale [Supporting Information],^17^ or Life Orientation Test‐Revised [LOT‐R; Supporting Information])^18^ were collected. The American Shoulder and Elbow Surgeons (ASES) functional outcome score was collected as well. For the purposes of this review, “preoperative resilience” refers to resilience scores obtained prior to surgery, whereas “concurrent resilience” refers to resilience scores obtained postoperatively during the recovery period.

Nonrandomized studies were evaluated for study quality using the Methodological Index for Non‐Randomized Studies score.^19^ Categories were scored as 0 (not reported), 1 (reported but inadequate), or 2 (reported and adequate). Noncomparative studies featured 8 questions for the Methodological Index for Non‐Randomized Studies score with total scores ranging from 0 (poor study quality) to 16 (high study quality), whereas comparative studies featured 12 questions with total scores ranging from 0 to 24.

### Statistical Analysis

Due to the heterogeneity of follow‐up duration, functional outcome scores, and clinical outcome measures of the included studies, the pooling of data was not pursued, and only a qualitative analysis was performed.

## RESULTS

**FIGURE 1:**
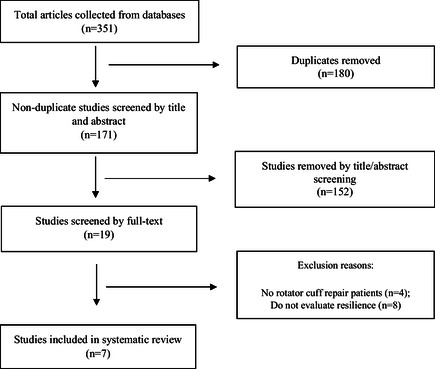
PRISMA diagram of screened studies. (PRISMA, Preferred Reporting Items for Systematic Reviews and Meta‐Analysis.)

A total of 351 records were identified, with 19 articles assessed by full‐text screening and 7 studies meeting the final inclusion criteria (Figure 1). All studies included were published between 2019 and 2024. Three of the included studies were retrospective cohort studies, and 4 were prospective cohort studies (Table 1). All studies investigated patients undergoing aRCR. The total number of patients included in the 7 different studies was 614 with sample sizes ranging from 48 to 131 patients. The average sample size across the studies was approximately 88.7 participants. Most patients were in their 50s and 60s with a mean age of 60.7 years (ranging from 55 to 62 years). Two studies only included patients with full‐thickness rotator cuff tears, whereas others reported varying proportions of partial‐ and full‐thickness tears, with full‐thickness tears ranging in size from small to massive.

**TABLE 1:** Study Demographics

Study	Level of Evidence	Sample Size (M:F)	Age (Yr)	Follow‐Up (Yr)	Tear Details	Repair Method	MINORS Score
Glogovac et al.^11^ 2019	IV	48 (25:23)	62 (34‐81)	0.5	All full‐thickness tears*	Double row	8
Hines et al.^14^ 2022	III	119 (71:48)	61 (51‐71)	1	63% small tears (<3 cm), 37% large tears (>3 cm)	Small tears: single‐row fixation	16
						Large tears: double‐row fixation	
Petrie et al.^6^ 2024	II	131 (57:74)	58 (48‐67)	2	NR	Double row	20
Porter et al.^5^ 2021	III	49 (28:21)	55 (47‐63)	4	55% full‐thickness, 45% partial thickness	Double row	16
Wilson et al.^12^, ^13^ 2023	II	91 (41:50)	62 (33‐80)	2.67	25% small, 32% medium, 15% large, 27% massive	Double row	19
Wilson et al.^12^ 2022	II	76 (NR)	61 (26‐80)	0.5	NR	Double row	16
Paul et al.^10^ 2024	III	100 (52:48)	60 (51‐69)	2	20% small medium, 61% large, 19% massive	Double row	18

*Note*: Age is presented as mean ± standard deviation (range).

M:F, male:female; MINORS, Methodological Index for Non‐Randomized Studies; NR, not reported; yr, year.

* Fifty percent supraspinatus only, 23% supraspinatus and infraspinatus, 15% supraspinatus and subscapularis, 8% supraspinatus infraspinatus and subscapularis, and 4.2% subscapularis only.

In accordance with the Sex and Gender Equity in Research guidelines, each included study was reviewed for sex‐disaggregated outcome analysis. Three studies incorporated sex as an independent variable in their analyses.^5^, ^10^, ^11^ Glogovac et al.^11^ found a significant association between sex and sleep quality (Pittsburgh Sleep Quality Index) at 6 weeks postoperatively, although this association was not observed at later follow‐up.^11^ Paul et al. and Porter et al. found no statistically significant differences in primary outcomes based on sex.^5^, ^10^ Two studies included sex in regression analyses and reported no significant associations.^6^, ^14^ The remaining studies reported sex distribution but did not perform sex‐disaggregated analyses of outcomes.^12^, ^13^

Five studies evaluated preoperative patient resilience using the BRS and its correlation with patient‐reported postoperative outcomes.^6^, ^10^, ^12^, ^13^, ^14^ One evaluated preoperative optimism using the LOT‐R scale and its correlation with patient‐reported postoperative outcomes.^5^ Lastly, 1 study evaluated preoperative resilience using the Connor‐Davidson Resilience Scale and studied its relationship with sleep quality using the Pittsburgh Sleep Quality Index.^11^ Four studies evaluated both preoperative and concurrent resilience and their relationship with postoperative outcomes, namely, the patient‐reported outcome ASES scale.^6^, ^10^, ^12^, ^13^

All 6 included studies that evaluated preoperative patient resilience (BRS scores: n = 5, LOT‐R scores: n = 1) and ASES scores found that preoperative patient resilience was not correlated with ASES scores 0.5 to 4 years following RCR (Table 2). Although Wilson et al.^13^ did not find preoperative BRS scores to be significantly correlated to postoperative ASES scores, the authors did find that the ASES function subscale score correlated to preoperative BRS scores (*r* = 0.223, *P* = .035) after a mean of 2.67 years.

**TABLE 2:** Effect of Preoperative and Concurrent Patient Resilience on ASES Scores

Study	Follow‐Up (Yr)	Preoperative Resilience vs ASES Correlation	Concurrent Resilience vs ASES Correlation
Hines et al.^14^ 2022	0.5 and 1	Nonsignificant at 6 mo (*P* = .97)	
Petrie et al.^6^ 2024	1 and 2	Nonsignificant at 1 (*R* = 0.15) or 2 yr (*R* = 0.07)	Nonsignificant between concurrent BRS scores and ASES function at 1 yr (*R* = 0.13) or 2 yr (*R* = 0.09)
Porter et al.^5^ 2021	Mean 4	No significant correlation with ASESf (*R* = 0.0972), ASESp (*R* = 0.1757), or ASES (*R* = 0.0169) at 4‐yr follow‐up	
Wilson et al.^13^ 2023	Mean 2.67 (range: 24‐47)	ASES: *r* = 0.156, *P* = .142	Significant correlations at mean 2.67‐yr follow‐up:
		ASESp: *r* = 0.069, *P* = .52	ASES: *r* = 0.291, *P* = .005
		ASESf: *r* = 0.223, *P* = .035	ASESp: *r* = 0.235, *P* = .036
			ASESf: *r* = 0.287, *P* = .006
Wilson et al.^12^ 2022	0.25 and 0.5	No significant correlations with preoperative ASES (*P* = .7293), 3‐mo ASES (*r* = 0.0414, *P* = .8313), or 6‐mo ASES (*r* = 0.1233, *P* = .6266)	No significant correlations with ASES scores at 3‐mo (*P* = .8223) or 6‐mo (*P* = .466) follow‐up
Paul et al.^10^ 2024	2	Multivariate analysis found that preoperative resilience was not associated with ASES score improvement (*b* estimate = −5.64; *P* = .150)	Multivariate analysis found that resilience at 2‐yr follow‐up was associated with ASES score improvement (*b* estimate = 6.41; *P* = .031)

ASES, American Shoulder and Elbow Surgeons; ASESf, ASES function; ASESp, ASES pain; BRS, Brief Resilience Scale; LOT‐R, Life Orientation Test‐Revised; mo, month; yr, year.

Four studies assessed the relationship between concurrent resilience (postoperative resilience measured at the same time as functional outcome scores, such as ASES) and ASES scores, with 2 studies showing significant correlation and 2 studies finding no significant correlation. Wilson et al.^13^ revealed a significant correlation between concurrent BRS and ASES scores at an average follow‐up of 2.67 years (*r* = 0.291, *P *= .005). Paul et al.^10^ also found that resilience measured at 2 years was significantly associated with improvements in ASES score (beta estimate = 6.41, *P* = .031) (Table 2). However, Petrie et al. and Wilson et al. found that concurrent resilience and ASES scores were not significantly correlated at 1 or 2 years (*P* = .658, *P* = .769) and 0.25 or 0.5 years (*P* = .822, *P* = .466) postoperatively, respectively.^10^, ^13^

Three of the included studies investigated postoperative outcomes based on different resilience categories (Table 3). They placed their patients into low‐, medium‐, and high‐preoperative‐resilience groups and had varying results. Petrie et al.^6^ found no significant differences in ASES score improvements at 1 or 2 years postoperatively across preoperative resilience groups. Similarly, Paul et al.^10^ reported no significant associations between preoperative resilience categories and ASES improvements at 2 years postoperatively. In contrast, Porter et al.^5^ found that patients placed in higher‐optimism cohorts showed significantly better functional and overall ASES scores at a mean follow‐up of 4 years (*P* = .05). In this study, the LOT‐R score, which quantifies optimism and pessimism as independent traits and has been shown to directly correlate with resilience, was used to assess psychological characteristics.^5^

**TABLE 3:** Resilience and Postoperative Outcomes by Resilience Category

Study	Follow‐Up	Change in Low‐Resilience Group	Change in Mid‐Resilience Group	Change in High‐Resilience Group	*P* Value
Petrie et al.^6^ 2024	1 and 2 yr	ASES 1 yr: +31.4	ASES 1 yr: +39.3	ASES 1 yr: +40.4	ASES 1 yr: *P* = .2256
		ASES 2 yr: +40.0	ASES 2 yr: +42.9	ASES 2 yr: +42.9	ASES 2 yr: *P* = .8478
		ASESf 1 yr: +9.8	ASESf 1 yr: +10.9	ASESf 1 yr: +11.5	ASESf 1 yr: *P* = .6587
		ASESf 2 yr: +12.0	ASESf 2 yr: +12.3	ASESf 2 yr: +13.2	ASESf 2 yr: *P* = .7690
Paul et al.^10^ 2024	**2 yr**	ASES: 47.0 ± 20.3	ASES: 45.0 ± 23.3	ASES: 38.2 ± 20.5	ASES: *P* = .406
		ASES*: 50.1 ± 30.1	ASES*: 46.5 ± 21.9	ASES*: 37.1 ± 19.4	ASES*: *P* = .175
Porter et al.^5^ 2021*	Mean 4 yr	N/A	ASESf: 34.99 ± 7.87	ASESf: 46.22 ± 1.49	ASESf: *P* = .048
			ASESp: 27.00 ± 6.44	ASESp: 44.47 ± 1.98	ASESp: *P* = .003
			ASES: 61.99 ± 14.08	ASES: 90.69 ± 3.12	ASES: *P* = .005

*Note*: All values are reported as mean ± standard deviation (SD) unless otherwise indicated. Statistical significance was set at *P* < .05.

ASES, American Shoulder and Elbow Surgeons; ASESf, ASES function; ASESp, ASES pain; yr, year.

* Subanalysis excluding patients with self‐reported mental health conditions.

All other findings relating patient resilience to less frequently reported outcome scores are reported qualitatively in Table 4.

**TABLE 4:** Qualitative Findings on Sleep, Mental Health, and Patient‐Reported Outcomes Across Included Studies

Study	Follow‐Up (Yr)	Qualitative Findings
Glogovac et al.^11^ 2019	0.5	There is a statistically significant but negligible predictive value (PPV) of CD‐RISC score on changes in PSQI score (*R* ^2^ = 0.009, *P* = .028) and nocturnal pain frequency (*R* ^2^ = 0.08, *P* = .041) at 6 mo; sex was also associated with PPV on PSQI but only at 6 weeks (*R* ^2^ = 0.016, *P* = .006)
Hines et al.^14^ 2022	**0.5 and 1**	The BRS score was not different between the tear size and SCB groups at any time point (*P* = .12)
Petrie et al.^6^ 2024	**1 and 2**	Higher‐rated resilience groups had higher VR‐12M scores at baseline (*P* = .0001), after 12 mo (*P* = .0002), and 24 mo (*P* = .0014)
Wilson et al.^13^ 2023	**2.67 (range in mo: 24‐47)**	BRS score correlates with PROM (*P* = .005) and SANE (*P* = .014)
Wilson et al.^12^ 2022	**0.25 and 0.5**	Moderate significant correlation between preoperative BRS and PROMIS‐10 after 3 mo (*P* = .009); moderate correlation between concurrent BRS and PROMIS‐10 after 3 and 6 mo of follow‐up (*P* = .0025); significant difference in physical and mental components of PROMIS‐10 at 3 and 6 mo between patients with and without mental health diagnoses
Paul et al.^10^ 2024	**2**	ASES and SANE scores did not differ among the resilience groups at 2‐yr follow‐up; low resilience patients had lower SANE scores at 2 yr compared with normal and high resilience patients (*P* = .019)

*Note*: Statistical significance was set at *P* < .05.

ASES, American Shoulder and Elbow Surgeons; BRS, Brief Resilience Scale; CD‐RISC, Connor‐Davidson Resilience Scale; mo, month; PROM, patient‐reported outcome measure; PROMIS‐10, Patient‐reported outcomes measurement information system global health‐10; PSQI, Pittsburgh Sleep Quality Index; SANE, single assessment numeric evaluation; SCB, substantial clinical benefit; VR‐12M, Veterans RAND 12‐Item Health Survey; yr, year.

## DISCUSSION

In this systematic review, we found that preoperative patient resilience was not associated with postoperative ASES scores after arthroscopic RCR. However, concurrent resilience measured during recovery showed significant correlations with ASES scores in some studies, suggesting a potential role for resilience in the postoperative period. Across 6 studies, there was no consistent association between preoperative resilience, as measured by the BRS or LOT‐R scales, and postoperative functional outcomes as measured by ASES. Wilson et al.^12^ reported the only significant correlation between preoperative BRS and postoperative functional outcomes was a significant correlation between preoperative BRS and ASES functional scores at a mean follow‐up of 32 months (*P* = .035). No other significant correlations between preoperative BRS and postoperative ASES scores were reported.

Of the 4 studies that explored the association between concurrent resilience and postoperative outcomes, only Wilson et al.^13^ and Paul et al.^10^ identified significant correlations between concurrent resilience and improvements in ASES scores, whereas Petrie et al.^6^ and Wilson et al.^12^ did not find a significant correlation between these variables. This indicates that resilience measured in the postoperative period may be more relevant to patient‐reported functional outcomes than preoperative resilience. This observation is consistent with findings from other orthopaedic surgeries in which resilience assessed postoperatively—whether during recovery^4^, ^7^ or at longer‐term follow‐up^3^—has been associated with patient‐reported outcomes. These data collectively suggest that the dynamic nature of resilience over the course of recovery may limit its utility as a sole preoperative prognostic parameter, helping to explain observed differences between preoperative and concurrent resilience findings.

Resilience is a dynamic construct that can change over time, particularly in response to stressors. For example, studies by Kalisch et al. and Köhne et al. showed that resilience levels can increase or decrease in response to significant external stressors, such as the coronavirus disease 2019 pandemic.^20^, ^21^ Paul et al.^10^ found that 38% of patients changed resilience categories throughout their recovery, further showing that resilience is malleable. This variability in resilience may be influenced by other psychological factors, such as self‐efficacy—an individual's confidence in their ability to successfully perform a specific task or behavior. Thomee et al.^22^ found that preoperative self‐efficacy regarding knee function was a significant predictor of postoperative physical activity levels and functional outcomes 1 year after anterior cruciate ligament reconstruction. This suggests that a patient's confidence in their ability to manage recovery may also affect their resilience. This may partially explain why concurrent patient resilience is more closely related to postoperative ASES scores than preoperative patient resilience scores.

Mental health outcomes in the context of orthopaedic surgery refer to patient‐reported measures of psychological well‐being, including depression, anxiety, life satisfaction, and perceived quality of life.^2^ These outcomes are closely linked to resilience, a dynamic construct reflecting an individual's capacity to adapt and recover from adversity. Konaszewski et al.^23^ showed that resilience significantly predicted mental health outcomes such as reduced depressive symptoms, greater life satisfaction, and improved well‐being in adolescents, underscoring its role as a protective factor across populations. Similarly, Drayer et al., in a study of military patients undergoing arthroscopic knee surgery, found that higher resilience scores were associated with better mental health–related quality of life, as reflected by higher Veterans RAND 12‐Item Health Survey and Patient Reported Outcomes Measurement Information System 43‐item scores, as well as superior knee function assessments at 6 months postoperatively.^24^, ^25^ These findings parallel results from several studies in our review, where higher resilience scores correlated with more favorable mental health outcomes both preoperatively and during follow‐up. Collectively, these data suggest that resilience may influence how patients cope with the emotional and psychological stresses of recovery and highlight the potential value of interventions aimed at strengthening resilience to optimize both psychological and functional outcomes after surgery.

Given the importance of psychological factors in recovery, concurrent resilience may be a more relevant indicator of postoperative outcomes than preoperative resilience. Although preoperative resilience did not correlate with functional outcomes in this review, resilience measured during recovery showed some association with postoperative ASES scores, suggesting that monitoring and supporting resilience throughout rehabilitation could be beneficial.

Future research should first aim to clarify the inconsistent relationship between resilience and postoperative outcomes observed in this review. In particular, prospective studies that assess resilience at multiple time points could help determine whether changes during recovery meaningfully influence functional outcomes. Standardizing resilience measurement tools would also improve comparability across studies and clarify its potential prognostic value. Additionally, exploring the interaction between resilience and other psychological factors, such as self‐efficacy, may help determine whether resilience functions as an independent predictor or is part of a broader recovery profile. Only if a consistent association is established should resilience‐targeted interventions, such as cognitive behavioral therapy or structured rehabilitation programs, be pursued to improve recovery after RCR.

### Limitations

This study has several limitations. The studies included in the review were heterogeneous in terms of follow‐up duration, functional outcome measures, preoperative versus postoperative resilience measurements, and tear characteristics, which prevented data pooling and meta‐analysis. Some included studies had only 6 months of follow‐up, which may not represent maximal recovery after aRCR and could underestimate or misrepresent the relationship between resilience and longer‐term outcomes.^25^ Additionally, most studies were nonrandomized retrospective cohort studies, introducing potential bias. The variability in resilience measurement and its inconsistent assessment across studies also limited the ability to draw firm conclusions about its role in predicting outcomes and conducting a pooled analysis. The methodological quality of included studies, as assessed by Methodological Index for Non‐Randomized Studies, was generally in the moderate range. Although some studies lacked features such as prospective sample size calculation, blinding, or control groups, these limitations are common in the available literature on this topic. Such design factors may have influenced the strength of observed associations and should be considered when interpreting the findings. Several studies did not control for important confounding factors such as comorbidities and psychological conditions, which could have influenced both resilience and postoperative outcomes. Lastly, Wilson et al.^13^ represent the same patient cohort, which may have introduced overlap in the data analyzed.

## CONCLUSIONS

Preoperative patient resilience does not correlate with postoperative ASES scores after aRCR. Concurrent patient resilience during recovery shows mixed associations with postoperative ASES scores, with some studies reporting significant correlations and others finding none.

## SUPPORTING INFORMATION

Additional supporting information can be found online in the Supporting Information section.

## DISCLOSURES

The authors (F.P.T., K.B.F.) declare the following financial interests/personal relationships, which may be considered as potential competing interests: F.P.T. reports a relationship with Trice Medical that includes equity or stocks; has patent with royalties paid to Tigon Medical; serves as a board or committee member of the American Board of Orthopaedic Surgery. K.B.F. reports a relationship with Liberty Surgical that includes paid expert testimony; reports a relationship with Vericel Corporation that includes speaking and lecture fees; reports a relationship with Medical Device Business Services that includes consulting or advisory; reports a relationship with DePuy Synthes that includes consulting or advisory; serves as a board or committee member of the American Orthopaedic Society for Sports Medicine. The other authors (P.S., N.(J.).O., R.W.P., J.T.W., F.J.S.) declare that they have no known competing financial interests or personal relationships that could have appeared to influence the work reported in this article.

## Supporting information

Supplementary Material
