# Supplementary material for: Preoperative Resilience Does Not Predict Functional Outcomes After Rotator Cuff Repair: A Systematic Review
Source: Arthrosc Sports Med Rehabil. 2026 May 7;8(2):e70025. doi: 10.1002/ars2.70025 (PMC13307219; doi:10.1002/ars2.70025)
Supplement: Supplementary file 1 — Supplementary Material [file ARS2-8-e70025-s001.pdf]

Supporting Information

Appendix:

1. Brief Resilience Scale[16]

Brief Resilience Scale (BRS)

	Please respond to each item by marking one box per row	Strongly Disagree	Disagree	Neutral	Agree	Strongly Agree
BRS 1	I tend to bounce back quickly after hard times	☐ 1	☐ 2	☐ 3	☐ 4	☐ 5
BRS 2	I have a hard time making it through stressful events.	☐ 5	☐ 4	☐ 3	☐ 2	☐ 1
BRS 3	It does not take me long to recover from a stressful event.	☐ 1	☐ 2	☐ 3	☐ 4	☐ 5
BRS 4	It is hard for me to snap back when something bad happens.	☐ 5	☐ 4	☐ 3	☐ 2	☐ 1
BRS 5	I usually come through difficult times with little trouble.	☐ 1	☐ 2	☐ 3	☐ 4	☐ 5
BRS 6	I tend to take a long time to get over set-backs in my life.	☐ 5	☐ 4	☐ 3	☐ 2	☐ 1

Scoring: Add the responses varying from 1-5 for all six items giving a range from 6-30. Divide the total sum by the total number of questions answered.

My score: ______ item average / 6

2. CD-RISC Questionnaire[17]

CD-RISC					
Entry	Not at all confident				Completely confident
1. I can adapt to change	0	1	2	3	4
2. I have a close, secure relationship.	0	1	2	3	4
3. Sometimes, fate or God can help me.	0	1	2	3	4
4. No matter what happens, I can cope with it.	0	1	2	3	4
5.Success in the past gives me confidence to face challenges.	0	1	2	3	4
6.I can see the humorous side of things.	0	1	2	3	4
7.Coping with pressure makes me feel powerful	0	1	2	3	4
8. After experiencing difficulty or illness, I tend to recover quickly.	0	1	2	3	4
9. There is always a reason why things happen.	0	1	2	3	4
10. No matter what the result is, I will try my best.	0	1	2	3	4
11. I can achieve my goal.	0	1	2	3	4
12. When things look hopeless, I don't give up easily.	0	1	2	3	4
13. I know where to go for help.	0	1	2	3	4
14.Under pressure, I can focus and think clearly.	0	1	2	3	4
15. I like to take the lead in solving problems.	0	1	2	3	4
16. I will not be discouraged by failure.	0	1	2	3	4
17. I think I am a strong man.	0	1	2	3	4
18. I can make unusual or difficult decisions.	0	1	2	3	4
19. I can handle unhappiness.	0	1	2	3	4
20. I have to act on my hunch.	0	1	2	3	4
21. I have a strong sense of purpose.	0	1	2	3	4
22. I feel in control of my life.	0	1	2	3	4
23. I work hard to achieve my goal.	0	1	2	3	4
24. I like challenges.	0	1	2	3	4
25. I am proud of my achievements.	0	1	2	3	4

3. Life Orientation Test Revised[18]

Revised Life Orientation Test (LOT-R)

Instructions:
Please answer the following questions about yourself by indicating the extent of your agreement using the following scale:

[0] = strongly disagree
[1] = disagree
[2] = neutral
[3] = agree
[4] = strongly agree

Be as honest as you can throughout, and try not to let your responses to one question influence your response to other questions. There are no right or wrong answers.

_____ 1. In uncertain times, I usually expect the best.
_____ 2. It's easy for me to relax.
_____ 3. If something can go wrong for me, it will.
_____ 4. I'm always optimistic about my future.
_____ 5. I enjoy my friends a lot.
_____ 6. It's important for me to keep busy.
_____ 7. I hardly ever expect things to go my way.
_____ 8. I don't get upset too easily.
_____ 9. I rarely count on good things happening to me.
_____ 10. Overall, I expect more good things to happen to me than bad.

Scoring:
1. Reverse code items 3, 7, and 9 prior to scoring (0=4) (1=3) (2=2) (3=1) (4=0).
2. Sum items 1, 3, 4, 7, 9, and 10 to obtain an overall score.

Note Items 2, 5, 6, and 8 are filler items only. They are not scored as part of the revised scale.
